# Towards an understanding of parietal mnemonic processes: some conceptual guideposts

**DOI:** 10.3389/fnint.2012.00041

**Published:** 2012-07-04

**Authors:** Daniel A. Levy

**Affiliations:** School of Psychology, The Interdisciplinary CenterHerzliya, Israel

**Keywords:** memory, parietal, fMRI, EEG, retrieval, recollection, familiarity, attention

## Abstract

The posterior parietal lobes have been implicated in a range of episodic memory retrieval tasks, but the nature of parietal contributions to remembering remains unclear. In an attempt to identify fruitful avenues of further research, several heuristic questions about parietal mnemonic activations are considered in light of recent empirical findings: Do such parietal activations reflect memory processes, or their contents? Do they precede, follow, or co-occur with retrieval? What can we learn from their pattern of lateralization? Do they index access to episodic representations, or the feeling of remembering? Are parietal activations graded by memory strength, quantity of retrieved information, or the type of retrieval? How do memory-related activations map onto functional parcellation of parietal lobes suggested by other cognitive phenomena? Consideration of these questions can promote understanding of the relationship between parietal mnemonic effects and perceptual, attentional, and action-oriented cognitive processes.

## Towards an understanding of parietal mnemonic processes: conceptual questions

A host of recent neuroimaging studies have documented the activation of areas of the posterior parietal cortex (PPC) during episodic memory retrieval (reviewed, *inter alia*, by Wagner et al., 2005; Skinner and Fernandez, 2007; Hutchinson et al., 2009; Spaniol et al., 2009; Kim, 2012). These findings converge with earlier electrophysiological studies that had reported event-related potentials recorded over parietal scalp that index episodic recognition (reviewed by Friedman and Johnson, 2000; Mecklinger, 2000; Rugg and Curran, 2007). One upshot of those neuroimaging studies is that medial parietal regions, including retrosplenial cortex, the precuneus, and posterior cingulate cortex, which are strongly interconnected with medial temporal lobe regions (Kahn et al., 2008), are activated in retrieval tasks; this accords with earlier clinical findings that damage to medial parietal areas may yield “retrosplenial amnesia” (Valenstein et al., 1987), a diagnostic category that should be understood as referring to memory impairments resulting from damage to the medial parietal regions in general (Aggleton, 2010). This interconnection has been more recently understood in light of all these areas forming part of the default mode network (DMN) (Raichle et al., 2001), which will be discussed below. Slightly more surprising in light of conventional neuropsychological wisdom were the reports of lateral parietal mnemonic activations. It is still unclear whether neuropsychological findings (e.g., Berryhill et al., 2007, 2009; Haramati et al., 2008; Simons et al., 2008, 2010; Schoo et al., 2011), are in consonance with the implication by neuroimaging of the parietal cortex in mnemonic processes, as lateral parietal damage does not seem to cause significant mnemonic impairments. However, even if intact lateral parietal cortices are not absolutely necessary for retrieval success, the nature of the lateral parietal activations engendered in connection with remembering is in need of explication. A number of explanations of lateral parietal mnemonic functions have been offered, but no single account offered to date seems to successfully explain the full range of empirical evidence, from a variety of paradigms, regarding this issue.

In the present article, I would like to examine several conceptual questions regarding relationships between memory, perception, attention, and action that are raised or emphasized by the parietal mnemonic issue, focusing specifically on findings regarding lateral parietal cortices. Several detailed scholarly reviews and meta-analyses of relevant studies have recently been published (e.g., Hutchinson et al., 2009; Uncapher and Wagner, 2009; Schoo et al., 2011; Kim, 2012), providing a database of findings upon which I will rely in framing the issues under examination. What follows is far from exhaustive, and reflects a mid-course effort to put some parts of the parietal mnemonic literature into a framework that might inform future empirical studies. At the risk of oversimplification, it might be heuristically helpful to consider the parietal mnemonic issue in light of a mosaic of several basic questions. These are: Do parietal mnemonic activations reflect memory processes, or the representation of the contents of memory? Are they indicative of pre-retrieval, retrieval, or post-retrieval processes? Are they related to the veridicality of memory? Are they primarily modulated by strength, quality (e.g., item vs. source), or quantity of retrieved information? Are they fundamentally lateralized or bilateral in nature? Do they represent parietal regions' membership in cognitive control, selective attention, or DMNs? In the following sections we will address these questions.

### Processes or contents?

Do mnemonically related activations indicate that lateral parietal cortices are loci of stored representations, or are they a substrate of intrinsic or ancillary retrieval processes? Current accounts of parietal mnemonic function may be categorized by how they answer that question. As the parietal areas that have been implicated in retrieval processes consist of a number of cytoarchitectonic and functional areas, multiple mnemonic functions are possible according to each account. The *Mnemonic Accumulator* model (Wagner et al., 2005; Donaldson et al., 2010) suggests that parietal activations during retrieval reflect the accumulation of a match signal between a recognition probe and representations stored elsewhere in the brain. The probe is judged to be old if that accumulated signal exceeds a certain threshold. Therefore, in this view, parietal mnemonic activations reflect processes intrinsic to retrieval. However, such signal accumulation is also posited to be part of perceptual judgment processes (Ploran et al., 2007), so the function is not specifically mnemonic. A related proposal is that inferior lateral parietal activation reflects expectations regarding the mnemonic status of a presented item, rather than sensitivity to the strength of a mnemonic trace (O'Connor et al., 2010; Buchsbaum et al., 2011). This *Expectation* approach, too, is a process function account.

The *Attention-to-Memory (AtoM)* account (Cabeza et al., 2008, 2011; Ciaramelli et al., 2008, 2010) explains parietal activations as reflecting attentional processes ancillary to retrieval: either top-down attention to cues preceding retrieval, or bottom-up capture of attention by retrieval output or the retrieval cue. In this account, activity in superior parietal cortices, ranging from the intraparietal sulcus (IPS) up to superior medial parietal regions, roughly the area of Brodmann Area (BA) 7, is posited to reflect top-down focal attention especially important for challenging retrieval tasks, while anterior inferior lateral parietal activations (in the vicinity of the supramarginal gyrus, overlapping BA 40) is explained as reflecting bottom-up capture of attention by easily accessed rich memoranda (Cabeza et al., 2008, 2011). In other words, this is a process account, but one that posits that the activations represent processes that are not intrinsically mnemonic.

In contrast to that attentional focus, posterior inferior parietal cortex in the vicinity of the angular gyrus (AG; BA 39) is not an area that has been implicated in attention systems, and therefore seems to require a different account—such as the *Cortical Binding of Relational Activation* (*CoBRA*) hypothesis (Shimamura, 2011; a related proposal is found in Vilberg and Rugg, 2008b), which focuses specifically on that region. In that model, ventral parietal cortex in the vicinity of AG is said to provide a stable substrate of representation for consolidated higher-level perceptual information—a content function.

Finally, in the *Output Buffer* (Vilberg and Rugg, 2007, 2008a,b, 2009; Guerin and Miller, 2011) and the *Memory-to-Action* models, (Haramati et al., 2008), ventral parietal areas are postulated to support temporary post-retrieval representations. The *Output Buffer* account, based on a similar role played by the Episodic Buffer, a proposed component of working memory (Baddeley, 2000), posits that left lateral inferior parietal cortex supports the representation or maintenance of retrieved episodic information—a content function. Proponents of this view (Vilberg and Rugg, 2009) complement the proposal by suggesting that more dorsal activations, especially in the middle (horizontal) segment of the left IPS, is responsive to the relative salience of retrieval cues associated with target information—a process function. The *Memory-to-Action* model (Haramati et al., 2008) is an extension of the *Output Buffer* model. Inspired by findings that extensive left ventral parietal lesions caused no impairment on recognition tasks that had yielded robust activations in those same areas, it postulates that ventral parietal areas are primarily important for maintenance of retrieved episodic information, in a format amenable to additional processing to guide subsequent behavior—a post-retrieval content function. In contrast to other models, the Memory-to-Action hypothesis postulates that ventral parietal activations represent post-retrieval processes exclusively, and therefore predicts that lesions in the areas identified by neuroimaging studies will not yield retrieval deficits *per se*, but may yield deficits in subsequent processing utilizing the retrieved information.

Among the process-focused accounts, the *Accumulator* account (Wagner et al., 2005; Donaldson et al., 2010) is based on a ramified model of recognition memory (Ratcliff, 1978; Ratcliff and Starns, 2009), and evidence that posterior parietal areas serve an accumulator function in perceptual decision-making (Ploran et al., 2007). What is said to be accumulated is a signal indicating “the amount of evidence that retrieval cue corresponds to a studied item” (e.g., Okada et al., 2012). However, this account seems to limit the parietal role in ecphory to recognition. Similarly, the *Expectation* account (O'Connor et al., 2010; Buchsbaum et al., 2011), which focuses on violation and confirmation of the mnemonic strength of the probe, would seem to be specific to the recognition process. This is problematic, as lateral parietal areas seem to be implicated in cued recall no less than in recognition. Because of methodological reasons, the majority of studies of parietal mnemonic function have employed recognition as the memory assay. However, cued recall studies extant in the literature (e.g., Allan and Rugg, 1997; de Zubicaray et al., 2007; Seibert et al., 2011a,b; Hayama et al., 2012; Okada et al., 2012) indicate that similar activations are found under those conditions as in recognition; we have recently confirmed that finding in an electroencephalographic (EEG) study of cued recall of cross-modal and unimodal pair associate learning (Levy, unpublished data). Since in successful cued recall there is no probe to be recognized (there is a cue, but in principle it may be stipulated to be old, and no judgment is made on it), accumulation of a probe-representation match signal is not task-relevant for retrieval success. Even if recognition of the probe does occur automatically, that is insufficient to successfully recall the target information. Such findings challenge the *Accumulator* and *Expectation* hypotheses. It has recently been proposed that the accumulator function should be assigned to mid-IPS regions (Suzuki et al., 2011; Okada et al., 2012), or more generally to dorsal parietal regions in BA 40 (Huijbers et al., 2010). Accordingly, the signal accumulation process and its IPS substrate might theoretically be partially dissociated from recollective and cued recall processes that lead to activations of AG. Weighing in against such a dissociation are findings of mid-IPS activation in cued recall (Okada et al., 2012). Furthermore, the finding of Guerin and Miller (2011) that activation in several parietal retrieval success areas (precuneus, AG, and IPS) track the strength of face picture memories rather than the decision criterion used in the memory task (in that study, a frequency judgment) may also be seen as challenging the assignment of an accumulator function to IPS.

One possible modification of the *Accumulator* model in light of the *CoBRA* account might provide a solution to a more general conundrum regarding recognition memory. Recognition requires the prior existence of neural ensembles representing target stimuli. Those ensembles were perceptually activated during acquisition, and achieve representational integrity—binding into a coherent stored representation of a perceptual experience—by Hebbian processes, initially mediated by the medial temporal lobes but later achieving independence through systems consolidation (Dudai, 2004). Recognition is executed by the comparison of such stored representations with a recognition probe. This putative process seems problematic: if the same neural ensemble that was activated during the perceptual acquisition of a memory trace of a given stimulus is also activated by a recognition probe (which is the identical or a very similar stimulus), how are the probe and trace ensembles simultaneously maintained for comparison? This is especially difficult to understand if the comparison occurs over time, as suggested by the *Accumulator* account, since that would seem to involve more complex comparative processes than basic repetition suppression implicated in perceptual priming (Henson and Rugg, 2003). *CoBRA* (Shimamura, 2011) posits that AG is a multimodal perceptual convergence zone supporting consolidated representations (but not representation during initial acquisition); successful recognition recruits the stored representations. Thus, cortical perceptual neural ensembles earlier in the perceptual stream can provide a substrate for a recognition probe, while the AG ensemble might represent the stored memory trace to which it is compared.

A related alternative is that AG might serve as a convergence zone for exogenous recognition probe perceptual inputs which activate more primary perceptual areas and endogenous representations provided by the hippocampus, in order to allow comparisons of perceived and retrieved representations. The degree of overlap between the representations could determine the degree of activation of the AG ensemble. That differential signal could be “read” by another neural ensemble (an accumulator ensemble) that is responsible for a judgment of recognition. That monitoring function might be the provenance of prefrontal areas assigned monitoring functions in mnemonic processes (Moscovitch and Winocur, 2002).

The *CoBRA* account stresses the ability of AG to support multimodal representations, but such a system could support unimodal recognition as needed. A recognition process that can take advantage of the multimodal integrative abilities of AG would provide the most versatile mechanism for ecological recognition. Furthermore, the ability to support integrative multimodal representations could provide the basis for recollective recognition, as well as of cued and free recall of unimodal or multimodal information.

MacKenzie and Donaldson (2009) note that parietal activations are often posited to reflect material-independent retrieval because they have been observed across stimulus types—words, line drawings, object pictures, landscape/object compound stimuli, and sounds. A similar notion is put forward by Donaldson et al. (2010), who go on to suggest that the parietal areas generically accumulate evidence rather than serving as a basis of representations. In contrast, the *CoBRA* account proposes that the lateral parietal regions are activated across material types not because they provide a signal accumulation function, but because of their multimodal integrative capacity.

Some support for the *CoBRA* concept is offered by a case study of a patient with cerebrovascular accident lesions limited to the vicinity of left AG, who was specifically impaired relative to controls in cued recall memory for cross-modal pair associate learning, while exhibiting intact performance on unimodal verbal pair associate learning (Levy, 2010).

The multimodality of representations in AG may explain the findings of Buchsbaum et al. (2011) in a study of recency effects in short-term verbal continuous recognition. They report that an area in middle inferior PPC which exhibited declining activity with increasing presentation-test lag was not active during verbal working memory maintenance, while such maintenance-related activity was found in the more anterior area Sylvian-parietal-temporal (Spt). This finding is taken as indicating that middle inferior parietal activations (themselves dissociated from a yet more posterior region identified with default mode activity) are unlikely to reflect retrieval-related maintenance as assumed by *Output Buffer* account. However, in Buchsbaum et al. (2011), the Spt area was identified using a Sternberg-type working memory task for letters, stressing verbal rehearsal and phonological characteristics, which is unlikely to activate the full range of multimodal stimulus features supported by AG as proposed by the *CoBRA* or *Output Buffer* accounts. Working memory maintenance for more complex stimuli, whether novel or drawn from long-term stores, might recruit AG as well.

In evaluating the *CoBRA* hypothesis, it must be noted that Sestieri et al. (2011), using a movie-based cued recollection paradigm which involves multimodal integration, report that AG was active during retrieval, but that same area was deactivated during a perceptual search task that used very similar materials, as would be expected by the inclusion of AG in the Default Mode Network (see below, “Cognitive Control, Selective Attention, or Default Mode?”). This would indicate that AG is not involved in the basic representation of perceptual information as suggested by *CoBRA*.

An alternative explanation of the activation of AG by retrieval is the *Memory-to-Action* hypothesis (Haramati et al., 2008), which, as mentioned above, is an extension of the *Output Buffer* hypothesis. *Memory-to-Action* is inspired by consideration of two processes in which parietal cortex has been shown to play an important role. The first is in the organization of information for the execution of serial tasks—the capacity which is impaired in ideomotor apraxia, a deficit often linked with left posterior ventral parietal damage (Wheaton and Hallett, 2007). The second point of departure is the complementary roles of parietal and hippocampal regions in navigation. In both animal and human studies, navigation based on allocentric spatial information has been linked to hippocampal substrates, while navigation based on egocentric spatial information has been connected with parietal cortices (Weniger et al., 2009). In fact, Weniger and colleagues (2009) report that in persons with unilateral parietal cortex lesions, egocentric memory expressed in navigation in a virtual environment is impaired, while allocentric, hippocampal-based navigation memory is spared. Importantly, in rodent studies it has been shown these two entities are not two separate representations, but reflect a transformation of allocentric to egocentric versions of spatial memory which enables the animal to move through the environment (Whitlock et al., 2008). In rodents, the hippocampus is primarily important for the representation of spatial information, but in humans its function seems to have developed to accommodate episodic information in general. The *Memory-to-Action* account speculates that the hippocampal-parietal translation of spatial information in rodents suggests hippocampal-parietal translation of episodic information in humans. Under ecological conditions, the function of retrieved memories is to support action in the environment. Remembering the identity, position, and temporal qualities of features of the world enables us to act efficiently. For this purpose, recognized and recalled items have more intrinsic value (and hence continue to be represented in the buffer) than stimuli that are judged to be novel, since known entities can constrain the degrees of freedom of potential action, while unknown entities provide less guidance for behavior. Similarly, high confidence memory judgments, being subjectively more reliable in guiding behavior, are more likely to lead to post-retrieval buffering than low-confidence judgments; this is in consonance with recent findings (Hayes et al., 2011) that ventral parietal cortex is more strongly activated by high- than by low-confidence endorsements, both for item memory and for item plus source memory. AG might provide a buffer dedicated to translation of memoranda to action-oriented representations. Aside from explaining the AG activations by episodic retrieval, this account addresses the findings that damage to lateral parietal areas do not generally cause mnemonic impairments in the same tasks that lead to the activation of those areas (Schoo et al., 2011). The process of recognition depends on other substrates; after recognition has happened, AG enters the process, so lesions would be expected to affect the subsequent use of retrieved information rather than the retrieval itself. Further research is required into parietal lesion effects on utilization of memory in guidance of subsequent action in order to assess the explanatory utility of this Memory-to-Action model.

This proposal converges with a recent account of the centrality of AG in semantic memory (Binder and Desai, 2011). Based on evidence they cite that AG responds strongly to concrete, high-frequency words and meaningful sentences, Binder and Desai (2011) conclude that the level of AG activation seems to reflect the amount of semantic information that can be successfully retrieved from a given input. Furthermore, they point out that, if considered as a convergence zone, “AG is notably bounded by dorsal attention networks that play a central role in spatial cognition, anterior parietal regions concerned with representation of action and posterior temporal regions supporting movement perception. This suggests that the AG may play a unique role in representation of event concepts” (Binder and Desai, 2011, p. 533). Supporting an action-oriented representation of information drawn from episodic memory may be yet another aspect of representational convergence afforded by such a transmodal association area (Mesulam, 1998).

### Timing: pre-retrieval, retrieval, or post-retrieval?

An additional taxonomy of accounts of parietal mnemonic function can be constructed using a temporal framework. For example, the implication of the *AtoM* account is that the dorsal and ventral parietal foci of retrieval-related activity should be found in two different time windows. The dorsal/superior parietal regions, implicated in focal top-down attention, need to be engaged preceding the actual retrieval, while the ventral foci centered around supramarginal gyrus, activated in bottom-up capture of attention, should come into play after retrieval. The *Accumulator* model and the *CoBRA* account focus on the moment of retrieval itself, for different reasons. The *Output Buffer* and *Memory-to-Action* accounts relate ventral parietal activations to post-retrieval processes, as should the *Expectation* account, since only after retrieval can the mnemonic status of an item be compared with one's expectation.

Evaluating the accounts based on temporal features of experimental data is challenging. Hemodynamic imaging does not have the temporal resolution required for adjudicating these claims, but EEG and magnetoencephalographic (MEG) studies can address the issue. The most recent studies to relate to this issue are those of Seibert and colleagues (2011a,b), in which MEG was recorded during cued recall following pair associate learning. Seibert and colleagues report very early activity—beginning within 100 ms of a retrieval cue and resolving in less than 400 ms—that distinguished correct living/non-living classification of the studied pair member of a presented cue from parallel correct classification of cues themselves. In their second study, taking advantage of the spatial resolution afforded by MEG, Seibert and colleagues (2011a) localize that activity to IPS. They conclude that this finding of very early activation associated with successful retrieval supports the *AtoM* account. It must be noted, though, that the classification responses in the task employed by Seibert and colleagues required retrieval of a limited type of gist information, and do not require the retrieval of the actual identity of the target memorandum. It remains to be demonstrated whether such early responses will characterize fuller episodic retrieval. Furthermore, the temporal differences between the conditions in that study were rather similar in IPL and SPL, contrary to the dorsal/ventral, top-down/bottom-up attention dissociation suggested by the *AtoM* account.

It is therefore instructive to examine earlier electrophysiological studies of episodic retrieval, which served as the original basis of claims for the existence of parietal mnemonic processes. The discussion of event-related potentials recorded over parietal scalp elicited in conjunction with successful recognition generally focuses on the 500–900 ms time range (Allan and Rugg, 1997; Friedman and Johnson, 2000; Rugg and Curran, 2007). The parietal components reach their strongest voltages later than those recorded over frontal scalp; they are also posited to reflect recollective richness in contrast to simple familiarity associated with the frontal foci (Rugg and Curran, 2007), although the interpretation of the frontal aspect is controversial (Voss and Federmeier, 2011). The question is whether that 500–900 ms time window is early or late, in the context of mnemonic processes. For example: in a simple word recognition task, in which the mean response time is 700 ms, the old/new ERP difference peaks at 600 ms on average, but extends from 400–900 ms (Johnson et al., 1998); can that ERP be conclusively identified as reflecting pre-retrieval, retrieval or post-retrieval processes? Furthermore, several studies have documented a late posterior negativity which extends several hundred ms after responses are made (e.g., Johansson and Mecklinger, 2003; Friedman et al., 2005; Herron, 2007; Mecklinger et al., 2007). This very robust activity, with old/new voltage differences sometimes several times stronger than differences in the window of earlier old/new parietal differences, is observed in tasks in which response conflict is potentially strong, or when additional information about the encoding episode (source memory) is to be retrieved. It may be argued that specifically those experimental conditions may serve as good models of ecological remembering, in which information may subjected to further analysis after its retrieval, in order to apply it to needs of the situation in which the rememberer is functioning. This contrasts with the standard serial recognition judgment paradigm in which activity is tracked in the laboratory, in which the task characteristics encourage the participant to cease processing the retrieved stimulus immediately after the recognition judgment in order to be ready for the next trial.

Thus, EEG and MEG studies have documented several time windows of parietal activation, which when taken together are compatible with the temporal characteristics of all of the extant accounts of parietal mnemonic activations. This suggests that parietal activations may represent a number of cognitive functions occurring during different epochs of retrieval processes, modulated by task demands. The implication of this distribution would be that several of the parietal mnemonic accounts might be valuable for understanding the activations in question, and they might therefore tile the time continuum rather than providing conflicting explanatory alternatives.

Another approach to determining the assignment of activations to slots in the sequence of retrieval stages is offered by the studies of Herron et al. (2004) and Vilberg and Rugg (2009). They employ a manipulation of the relative probability of old and new items in blocks of the test battery, for item recognition in the former study and for source judgments in the follow-up study. The assumption of that manipulation is that “neural activity can only vary according to the relative probability of old and new items after the items have been identified as such” (Vilberg and Rugg, 2009), and modulation by probability indexes post-retrieval processes, but not retrieval itself. Vilberg and Rugg (2009) report that two superior parietal regions, one anterior and one posterior to mid-IPS, showed such probability-sensitive retrieval success effects. In contrast, source-retrieval-related activations in ventral regions in the vicinity of AG and mid-IPS activation irrespective of source accuracy were insensitive to old/new probability differences. This represents a “process-dissociation” approach to unraveling the temporal structure of parietal mnemonic activations.

An extension of the temporal taxonomy is the question of the relationship between parietal retrieval effects and parietal encoding effects that have been investigated in other studies. Notably, it has been reported that 85% of the positive subsequent memory effects in the lateral PPC occurred in superior parietal regions (in the IPS or BA 7 dorsal to it), while activation in more ventral parietal areas during encoding predicts subsequent forgetting (reviewed by Uncapher and Wagner, 2009; Kim, 2011). This distribution of effects has been explained in terms of attention: top-down attention during encoding, supported by superior parietal substrates, yields more effective encoding and better memory, while the capture of attention by non-target stimuli in the environment, or DMN-related mind wandering, leads to less effective encoding and so to poorer subsequent memory (Uncapher and Wagner, 2009; Kim, 2011). Such findings and explanations are differentially challenging for the various accounts of retrieval effects. In the accounts that assign parietal activations to attentional or signal accumulator processes, parietal retrieval effects may be completely orthogonal to encoding effects. However, the contrast between the existence of retrieval success and the lack of subsequent memory effects in AG is a challenge to the *CoBRA* account, and to the *Output Buffer* hypothesis as well. If parietal mnemonic activations at retrieval reflect memory contents rather than memory processes, we might expect greater encoding-retrieval overlap than is reported. This would certainly be the case according to the view that an act of remembering, especially episodic remembering, consists of the coordinated reactivation of sensory/perceptual regions that were activated at the time of encoding (e.g., Squire, 1987; Wheeler et al., 2000). Studies using fMRI have provided evidence supporting this reactivation/reinstatement hypothesis (e.g., Johnson and Rugg, 2007; Kim et al., 2010; and other studies reviewed by Danker and Anderson, 2010). If AG plays a content role, as suggested by *CoBRA*, we should expect to find encoding-retrieval overlap. However, this has not been reported; indeed, in one study, higher-levels of AG activation during the presentation of task-irrelevant face pictures were correlated with subsequent failure to recognize those faces (Minamoto et al., 2012). That heightened activation in ventral parietal regions during encoding leads to subsequently poorer memory is in accordance with the *AtoM* account: because items to be encoded are ostensibly in the focus of attention, during encoding there is typically no need for attention reorienting. Thus, VPC activity at encoding may reflect bottom-up attention to task-irrelevant stimuli or thoughts, and hence predict encoding failure (Daselaar et al., 2009).

An additional temporal frame issue poses another challenge to one feature of the *CoBRA* account. According to *CoBRA* (Shimamura, 2011), AG is especially important for supporting consolidated episodic representations, which at an earlier stage in their lifespan are more dependent on the hippocampus. Such systems consolidation (Dudai, 2004) of memory from initial medial temporal lobe representations to posterior cortical substrates has indeed been posited by many memory theories. However, the time frame of systems consolidation in humans may range from days to decades—while AG activations during retrieval have been reported when retrieval follows encoding by less than an hour. This seems to be problematic for that aspect of the *CoBRA* hypothesis.

### Veridical or asserted memory?

The various accounts of parietal mnemonic activations may also be assessed on the basis of how well they fit in with the reports that lateral parietal activity is correlated with the subjective impression that an item is old, such that it is found in false alarms more than in correct rejections (Wheeler and Buckner, 2003; Kahn et al., 2004; Shannon and Buckner, 2004; Wagner et al., 2005). This is most problematic for the *CoBRA* account, since that proposal relates the activations to the existence of representations in cortex, which theoretically should not exist for foil probes. Subjective memory activations require the *AtoM* account to incorporate the capture of attention not by mnemonic representations themselves (since there are no such representation in the case of false alarms) but by the cues that are used to probe it. It also requires the *Accumulator* hypothesis to accept that the signal cannot be the raw comparison of probe to representation, but rather the output of an earlier process that has already made that comparison, and requires the *Output Buffer* hypothesis to allow the storage in the buffer of a probe-related representation rather than retrieved information alone. The activation by subjective judgment is least problematic for the *Memory-to-Action* hypothesis, as it posits that a post-retrieval-decision trace is what is held in a buffer for further processing, whether it is veridically old or not. It is notable in this regard that neuropsychological findings have led some researchers to suggest that parietal cortex does not directly participate in retrieval, and instead reflects the subjective experience of recollection (Ally et al., 2008).

In a related vein, O'Connor and colleagues (2010), using a Posner cueing paradigm adapted for a recognition memory, show that ventral parietal activation (in both the AG and supramarginal gyrus) is more sensitive to expectation of whether an item would be old or new than to whether it was actually old or new. Such expectation judgments are theoretically orthogonal to the veracity of the mnemonic judgment. However, the trial-by-trial cueing in that study changes the cognitive nature of the task, and focuses the participant on constant evaluation of the cue-stimulus relationship, and therefore it is uncertain whether that pattern of findings readily generalizes to ordinary retrieval task processes.

### Quantity, quality, or strength?

It has been observed that “AG retrieval-related activity has been reported to covary with the amount of information recollected” (Hayama et al., 2012), supporting a content approach to understanding such activations. However, determining whether particular experimental conditions yield differences in ecphoric quantity or quality is not trivial. For example, Guerin and Miller (2011) describes the difference between memory for stimuli presented 1–2 times and stimuli presented 5–6 times as being “the amount of information recollected,” and suggests interpreting recognition-related activations which they found in AG accordingly. But does stimulus repetition necessarily lead to quantitative information differences? Even if repetition increases the *likelihood* of recognition (and therefore of measures of performance such as percent correct scores), it may not increase the *amount* of information available for each individual recognized stimulus. Of course, interpretation of repetition effects is a function of what one means by the “amount of information.” If one is given the task of remembering object pictures, e.g., successful encoding of the name of the object or basic aspects of its appearance would enable recognition in contrast to foils that are other objects, but not necessarily in contrast to foils that are minimally modified versions of the original object. The additional details required for the latter task might best capture the idea of “amount of information” about a recognition target, and their acquisition might be a function of repetition. This does not seem to have been investigated.

Suzuki and colleagues (2011) employed encoding repetitions, but understand them as affecting memory strength rather than quantity of information. They conducted fMRI while participants made responses indexing the initial, second, third or fourth appearance of object pictures. They report that while mid-IPS activation increased linearly with the degree of repetition, no such effect was found in more ventral parietal areas. They interpret that absence of activation as possible evidence that the repeatedly presented items elicited little or no recollection. However, the authors do not report whether retrieval success effects were not found at all in VPC, or whether they were found, but not repetition-graded.

Another approach to operationalizing memory strength is based on ratings of confidence in mnemonic judgments. Hayes and colleagues (2011) contrasted differences in confidence during tasks requiring item or source retrieval, and report that parietal mnemonic activity tracked confidence ratings, with dorsal areas showing low-confidence activity and anterior ventral areas in the vicinity of BA 40 showing high-confidence activity in both tasks. This finding is offered in support of the *AtoM* account.

Turning to qualitative distinctions, source memory retrieval success is often offered as evidence for qualitative differences in memory processes (e.g., within a dual-process framework; Yonelinas, 2002). However, source memory judgments employed in parietal mnemonic studies are generally binary (e.g., which task—pleasantness or concreteness judgment—was used for encoding this stimulus? Did this stimulus previously appear on the right or left of the screen? Was it presented in red or in green?). Few studies provide multiple opportunities to retrieve information about the encoding episode—which could provide a quantitative rather than qualitative characterization of memory for retrieval incident.

Furthermore, and in connection with the above-mentioned proposal that AG might serve as a convergence zone for exogenous recognition probe perceptual inputs in order to allow comparisons of perceived and retrieved representations, is a key and often overlooked point regarding experimental studies of recognition. Although they are sometimes purported as being able to dissociate familiarity from recollection (Yonelinas, 2002), recognition tests are nevertheless invariably tests of *contextual* recognition, which is problematic for the assessment of familiarity. Certainly in recognition tests employing verbal materials, and even for recognition tests using novel visual or auditory stimuli, participants are seldom if ever asked if a probe stimulus is at all familiar—whether they have ever experienced it at any point in the past. Invariably, the test question always is: Did you experience this stimulus *in the encoding episode*? Thus, the standard recognition memory experiment does not identify processes supporting simple familiarity, but rather processes enabling the linkage of a stimulus with a particular spatio-temporal context. Context is a multisensory entity; accordingly, if AG provides a multimodal convergence zone, it has added value for recognition not in representation of the probe or target stimulus, but vis-à-vis the context in which it was experienced. Therefore, remember-know response types may index differences in degree of memory strength rather than in categorical differences.

### Lateralized or bilateral?

Two domains have been noted in which lateralization of parietal mnemonic activations require consideration: material type specificity (e.g., verbal vs. visual stimuli) and retrieval-type specificity (recognition vs. cued recall).

Left-lateralized PPC activity has been observed for non-verbal information (faces) and non-visual information (Guerin and Miller, 2009), words, pictures, and sounds (Shannon and Buckner, 2004). It should be noted that the effect lateralization may be relative, as a function of the activation threshold selected for report. For example, in Shannon and Buckner (2004), parallel (albeit weaker) activations are reported in the right parietal areas activations for the same stimuli that activated the left hemisphere. For non-Western, non-verbal music clips assumed to be encoded and represented primarily by higher-order auditory regions in the right-hemisphere successful retrieval effects were observed only in the right PPC (Klostermann et al., 2009). It is possible that the dominance of left-lateralized effects reflects the use of left hemisphere verbal or semantic retrieval processes (Shimamura, 2011), perhaps even for materials such as faces, for which right-hemisphere perceptual representations are dominant, but for which descriptive heuristics may be employed as part of a recognition strategy.

Left lateralization of ventral parietal activations is problematic for the *AtoM* account, as much recent evidence indicates that non-spatial attentional abilities such as detection of behaviorally relevant and novel stimuli and reorienting to stimuli in either visual field that are presented outside the focus of attention (stimulus-driven reorienting) recruit a right lateralized ventral attention network (Corbetta and Shulman, 2011). It would therefore be expected that reorienting of attention to retrieved information on the basis of its salience (Cabeza et al., 2008, 2011) would lead to stronger right-hemisphere activations, irrespective of material type. Content accounts of parietal mnemonic activations are more compatible with material-specific or semantically driven lateralization.

In contrast to the left-lateralization of parietal recognition-related activations, event-related potential, and hemodynamic imaging studies of cued recall sometimes report more bilaterally distributed parietal activations (e.g., EEG: Allan et al., 1996; Allan and Rugg, 1997, but not Donaldson and Rugg, 1999; fMRI: Schott et al., 2005; Hayama et al., 2012 but not de Zubicaray et al., 2007), and we have found this to be the case for EEG recorded in conjunction with cued recall following audio-visual pair associate learning as well (Levy, unpublished data). This might account for the finding by Davidson et al. (2008) that in patients with PPC lesions (four left- and one right-hemisphere lesions), impairment was observed on an old/new recognition test and patients offered fewer “remember” and more “know” responses than did controls. In contrast, the patients were not significantly impaired on the cued recall or source memory tests. Since cued recall has been reported to have a more bilateral activation distribution, compensation by the intact hemisphere might have been more effective specifically for that task. As noted by Hayama et al. (2012), this distribution might also explain the lesion data of Simons et al. (2010).

### Cognitive control, selective attention, or default mode?

#### Default mode

An important recent concept in cognitive neuroscience is the functional parcellation of the brain into networks supporting externally oriented active perception and goal-directed cognition and networks supporting internally oriented mentation, with the latter often referred to as the (DMN; Raichle et al., 2001). Noting the overlap between membership of the parietal aspects of the “retrieval success network”—AG, posterior cingulate cortex, and the precuneus—and their membership in DMN, Kim (2012) suggests that the optimal approach to understanding retrieval-related activations in AG is in terms of its belonging to DMN. Similarly, Kim (2012) suggests that the dorsal parietal mnemonic activations should be understood in terms of those areas being part of the cognitive control network, along with a range of prefrontal regions.

However, some recent findings seem to vitiate the explanatory power of the default mode account for AG-focused old-new effects. In a study by Sestieri et al. (2011), participants performed cued recollection of details of previously movie scenes, arguably a strong model of ecological recollection. They report dissociation within the DMN areas between AG which was implicated in such retrieval processes, and mPFC, which was not so involved. Similarly, Suzuki et al. (2011) report dissociation in repetition effects between the precuneus, which like IPS showed graded increase with repetition, and hippocampus and retrosplenial/posterior cingulate cortex, which showed a graded decrease of activation. Reas et al. (2011) report that in the course of attempted recall of strongly and (especially of) poorly remembered word-pair associates, participants exhibited deactivation in left inferior parietal lobe in the vicinity of AG (along with anterior hippocampus and other aspects of DMN). This is very much the opposite of what might be expected on the basis of prior studies that report that the degree of AG activation increases with greater recollective strength, from a form of retrieval that would seem to require the greatest degree of recollection. Furthermore, in an experiment that tracked the effects of word study-test lag on retrieval-related activations, Buchsbaum et al. (2011) found that both a medio-lateral inferior parietal area that showed decreasing activation with longer lag, and an anterior temporal-parietal region abutting the Sylvian fissure implicated in basic verbal working memory rehearsal, were functionally and anatomically dissociable from a third, more posteriorly situated, parietal area identified with DMN. Such divergent dissociations seem to indicate that explanations of lateral parietal activations simply in terms of general DMN processes may not be an effective approach.

#### Cognitive control

Consideration of the second putative network implicated in parietal mnemonic activations, which Kim (2012) labels the cognitive control network, intuitively brings to mind the *AtoM* hypothesis (Cabeza et al., 2008, 2011; Ciaramelli et al., 2008, 2010). That account posits that dorsal parietal activations reflect allocation of attention to memory search. Understandably, to the extent that superior parietal lobes are a substrate of top-down attention processes (Corbetta and Shulman, 2002, 2011), it is to be expected that just as in any cognitive process, attending to the task will bring greater chances of its successful execution. This proposal is supported by findings that more challenging instances of successful recognition—e.g., recognition judgments which are identified as reflecting familiarity rather than recollection, or those with low-confidence—are more likely to be associated with superior parietal activations than recollective, high-confidence, ostensibly less effortful recognition. Additionally, DPC activity has been found to decrease across repeated retrieval attempts (Kuhl et al., 2007).

Recently, Ciaramelli et al. (2010) have noted that dorsal parietal activations—specifically in the IPS—are associated with trials in which probes are preceded by cues that could initiate retrieval before probe presentation. However, in that study, the cues were all studied words in themselves, so the evidence accruing from it that activation reflected orienting rather than automatic retrieval is equivocal.

Counter to the *AtoM* claims that dorsal parietal activations are purely attentional is the integrative analysis reported by Hutchinson et al. (2009), indicating that foci of dorsal parietal activation in studies of recognition do not completely overlap the foci of activations from studies of visual attention and working memory. Hutchinson and colleagues report that top-down attention foci are mostly to be found in the medial bank and posterior portion of IPS, and in SPL, while retrieval success activations lie lateral to most attentional foci. However, Hutchinson and colleagues acknowledge that divergence may represent the specific types of visual-spatial attentional foci that they compiled for comparison, whereas recognition memory tasks might recruit slightly different attentional processes. A recent specific examination of the dorsal parietal activations by Sestieri and colleagues (2010), in which retrieving remembered details of a viewed video clip was contrasted within subjects with perceptual search of the same kinds of details, yielded IPS activations that not only did not overlap, but actually suggested competition between attentional and mnemonic processes.

There are, of course, other cognitive processes other than purely attentional ones with which dorsal parietal mnemonic activations might be linked. Kim (2012) notes that across studies, components of the cognitive control network are activated more strongly by instances of source retrieval than of item retrieval. Among the possible reasons for that difference is the fact source memory judgments are generally made between two alternatives. A generate and test approach can be used in order to weigh the relative similarity of each of the possible representations compared with a stored representation. Thus, source judgments may use working memory, in which superior parietal cortex is strongly implicated (Wager and Smith, 2003) to represent the alternatives and judge between them, while in item recognition the entire probe is perceptually available until judgment.

Another cognitive control distinction, suggested by Kim (2012), is that iterative searches and verification of retrieved information may engage more consistently during a hit than during a correct rejection of an unstudied probe. However, it seems that at least iterative searches must be more part of the correct rejection process than of the recognition hit process, just as in visual search tasks reports of target presence must be faster on the average than reports of absence. An alternative is provided by the *Expectation* account, based on the study of Herron et al. (2004), who showed that areas implicated in cognitive control, but not default mode areas, retrieval success effects decreased or even reversed when old/new stimulus ratios increased from 25 to 75% of the test probes. Sensitivity to probability reflects an executive function/cognitive control account which is applicable not just to memory judgments, but to perceptual decision-making in general, as indicated by the study of Ploran et al. (2007). The involvement of such processes in mnemonic judgments and their independence from purely selective attention processes may be related to the report of Vincent and colleagues (2008) of three networks dissociated by resting-state connectivity, which they identify as representing dorsal attention, fronto-parietal control, and hippocampal-cortical memory systems. These systems occupy a progressively superior-rostral to inferior-dorsal swath along lateral parietal cortex. The fronto-parietal control aspect of these networks seems to overlay the convergence maps of retrieval success and recollection effects provided by Hutchinson and colleagues (2009). It therefore seems appropriate to conduct additional parietal mnemonic studies tracking the impact of the range of factors implicated in strategic “working-with-memory” processes on activations in the midrange parietal areas directly inferior to mid-IPS.

#### Selective attention

The *AtoM* account's attempt to interpret ventral parietal activations in terms of attentional processes—as representing “reorienting of attention to internal representations”—seems somewhat more problematic than the attentional account of dorsal parietal activations. In the integrative analysis of Hutchinson and colleagues (2009), the divergence between memory- and attention-related activations in ventral PPC is quite strong. More recent studies with a higher spatial resolution (e.g., Sestieri et al., 2011) confirm that lack of concordance. The real difficulty with the *AtoM* account, though, is conceptual. What might it mean “to orient attention toward internal representations” in the context of a probe-driven recognition task? Ciaramelli et al. (2010) frame the *AtoM* claim by focusing on non-cued and invalidly cued recognition trials (i.e., recombined pairs), for which activation was found in the AG. However, that operationalization may not capture orienting, but rather the need for recollective processes that are recruited for recognition of a probe when it is not supported by its study context (Tibon et al., 2012).

In a recent study, Cabeza and colleagues (2011), attempted to address the attention-mnemonic foci overlap discrepancies, and address the conceptual issue of nature of orienting in the context of retrieval. Participants learned progressive word-pair chains constructed on the basis of serial semantic associations. They then were presented with the initial word of such a chain and asked to recall the second member of the third linked pair in that chain. This retrieval condition was contrasted with a task of similar duration, in which participants monitored a stream of letters and noted the appearance of a vowel. The authors propose that the initial stimulus in each case requires orienting to the task case, while the appearance of the vowel target or the retrieval of the target word were both incidents of detection. Overlapping (but not identical) activations were found in dorsal parietal regions for the orienting aspects of both tasks, while overlapping activations in ventral parietal regions were associated with the detection phases. Furthermore, in ROI analyses, the parietal areas showing greater mnemonic detection activation were functionally connected with MTL, while those showing greater perceptual detection activation were functionally connected with visual areas. Cabeza and colleagues (2011) argue that these findings suggest that ventral parietal activations associated with target detection are attentional in nature. However, in this paradigm, detection is stressed at the expense of retrieval. Since the target word was the third retrieval in a chain, it was characterized not only by retrieval processes (in which functional connectivity with MTL is appropriate) but by the fact that a target was identified. Significantly, the authors note that the ventral parietal activations did not differentiate between successful and unsuccessful retrieval when examined in a whole-brain analysis. This contrasts with the cases in which studied stimuli yield ventral parietal activations even when they are not the cases to be endorsed (Shannon and Buckner, 2004; Donaldson et al., 2010). Therefore, that study does not necessarily aid characterization of the specifically mnemonic processes in which ventral parietal areas are implicated. Accordingly, the intriguing concept of orienting to internal representation seems to require further explication.

## Conclusion

As we have seen, despite the wealth of studies that have been conducted about lateral parietal involvement in long-term episodic memory, uncertainties still abound. Some of the accounts mentioned above maintain that there is nothing specifically mnemonic about parietal activations, but rather that they reflect general purpose attentional or control processes that can support a wide range of cognitive abilities. In other accounts, parietal activations during episodic retrieval are held to reflect aspects of perceptual representation. The interpretive dichotomies of the preceding sections are offered as a heuristic for consideration of the wealth of evidence that has become available regarding this issue. Considered synoptically, they suggest that future research should be oriented toward revealing the mosaic of dimensions characterizing parietal mnemonic processes: delineating subareas (including laterality) and time windows; expanding the range of material types examined; and most importantly—using more ecological assays of memory that can reveal the complex cognitive interactions that may characterize the intersection of perceptual, attentional, mnemonic, and action processes that represent parietal contributions to remembering.

### Conflict of interest statement

The author declares that the research was conducted in the absence of any commercial or financial relationships that could be construed as a potential conflict of interest.
